# Effects of Prenatal Exposure to Pollutants on Children’s Development: Additional Issues

**DOI:** 10.1289/ehp.11763

**Published:** 2008-10

**Authors:** José G. Dórea

**Affiliations:** Faculty of Health Sciences, Universidade de Brasilia Brasilia, Brazil, E-mail: dorea@rudah.com.br

Tang et al. (2008) made a significant contribution to understanding the effects of pre-natal exposure to coal-burning pollutants:

Decrements in DQs [developmental quotients, measured by the Gesell Developmental Schedules] were significantly associated with cord blood levels of PAH–DNA adducts and lead, but not mercury.

Recent developments compel us to consider Hg sources and attenuating factors in neurodevelopment related to early human exposure. My comments are specifically directed to breast-feeding (neurodevelopment modulator) and uncontrolled sources of Hg: ethylmercury (EtHg) in thimerosal-containing vaccines (TCV), and methyl-mercury (MeHg) consumed in rice—not fish. Breast-feeding is essential to promote or prime neonatal neurodevelopment, and China is among the countries that use TCV; therefore, controlling for these variables is important in neurodevelopmental studies (Dórea 2007a).

Tang et al. (2008) discussed the literature showing that prenatal Hg exposure is related to adverse neurodevelopmental outcomes at 2 years of age. During the post-natal period, the central nervous system is still vulnerable to Hg exposure; therefore, additional early exposure to Hg may be difficult to disentangle from prenatal events. Newborns in China are immunized with TCVs carrying concentrations of Hg ranging from 12.5 to 17.5 g Hg/dose (Dórea 2008). Furthermore, TCVs, such as the hepatitis B vaccine, are given immediately after birth. Also, some mothers could use products containing thimerosal during pregnancy (e.g., Rh-negative mothers taking anti-RhoD immune globulins). Considering that Tang et al. (2008) reported a 70-day range in gestational age for their cohort, it is reasonable to speculate that if TCV could be taken during the first 10 weeks postnally, EtHg exposure should be normalized. We were not informed of the immunization schedule (or maternal exposure to thimerosal products) of this cohort, but it is possible that in the time interval of gestational-age variation (10 weeks), there would be opportunity for five shots of TCV (Dorea 2007a). Considering the reported 70-day interval of gestational age, we should expect an even wider range of Hg exposure (on a body mass basis) due to variation in birth weight and respective rate of weight gain.

The effects of TCV-EtHg exposure on neurodevelopment are controversial. The most recent epidemiologic studies (Thompson et al. 2007; Young et al. 2008) exemplify current uncertainties related to the U.S. Federal Court compensation claimed on adverse effects triggered by TCVs (Offit et al. 2008). Although the statistical analysis of Tang et al. (2008) was well designed for prenatal events, perinatal TCV-EtHg exposure not evaluated by cord blood measurements could not account for effects (albeit transient) on neurodevelopment at 2 years of age.

Studies that measured neurodevelopmental outcomes as a result of prenatal exposure to neurotoxic substances have shown that breast-feeding, in most cases, can counteract some of the adverse effects (Dórea 2007b); compared with formula feeding, children had better neurobehavioral scores due to prenatal exposure to several classes of environmental pollutants. Because breast-feeding is an important modifier of neurodevelopmental outcome, not controlling for its duration could be a limitation in the Gesell Developmental score (GDS) outcomes related to Hg. Indeed, using principal component analysis, we have found effects of prenatal and postnatal Hg from both EtHg (from vaccines) and MeHg (fish consumption) in exclusively breast-fed children that were also evaluated by GDS (Marques et al. 2008). Tang et al. (2008) showed that cord blood Hg was three times lower than that reported for the Faroe Island whale-eaters, thus attributing the 7.0 μg Hg/L to low fish consumption (only nine mothers consumed fish or shell fish); compared with the high concentrations of Hg in whale meat, the Hg levels in these non–fish-eating mothers are relatively high. In this context, it should be noted that recent studies have indicated that rice can significantly contribute to MeHg exposure in China (Feng et al. 2008; Qiu et al. 2008). A post hoc assessment of these issues can enrich Tang et al.’s study.
